# Epidemiologic and Genomic Investigation of Sexually Transmitted *Shigella sonnei*, England

**DOI:** 10.3201/eid3107.241584

**Published:** 2025-07

**Authors:** Hannah Charles, David R. Greig, Craig Swift, Israel Olonade, Ian Simms, Katy Sinka, Kate S. Baker, Gauri Godbole, Claire Jenkins

**Affiliations:** United Kingdom Health Security Agency, London, UK (H. Charles, D.R. Greig, C. Swift, I. Olonade, I. Simms, K. Sinka, G. Godbole, C. Jenkins); University of Liverpool, Liverpool, UK (D.R. Greig, K.S. Baker, C. Jenkins); University of Cambridge, Cambridge, UK (K.S. Baker)

**Keywords:** *Shigella*, *S. sonnei*, *S. sonnei* blaCTXM-15, sexually transmitted infections, antimicrobial resistance, enteric infections, bacteria, shigellosis, England

## Abstract

Shigellosis is a bacterial infection that causes enteric illness and can be sexually transmitted, particularly among gay, bisexual, and other men who have sex with men. Multiple extensively drug-resistant *Shigella* strains have been detected through genomic surveillance and are associated with plasmids carrying the gene variant *bla*_CTX-M-27_ in the United Kingdom. We report an increase in possible sexually transmitted cases of *Shigella* bacteria carrying the *bla*_CTX-M-15_ gene variant, which was previously associated with travel. In 2023, there were 117 cases belonging to the 10 single-nucleotide polymorphism linkage cluster t10.1814. Although this cluster has been documented in England since August 2019, genetic analyses revealed that the *bla*_CTX-M-15_ gene variant entered the lineage on a novel resistance plasmid coinciding with the first outbreak case. Our analysis highlights the shifting antimicrobial resistance landscape of sexually transmitted *Shigella* bacteria. Parallel emergence of resistance determinants against third-generation cephalosporins in sexual transmission networks suggests high levels of antimicrobial selection pressure.

Shigellosis is a gastrointestinal infection caused by 1 of 4 bacterial species, *Shigella sonnei*, *S. flexneri*, *S. boydii*, or *S. dysenteriae*. Common symptoms include bloody diarrhea, abdominal pain, cramps, fever, nausea, and vomiting (*1*). *Shigella* spp. are anthroponotic and transmitted by fecal–oral contact (*1*), from hands or objects that were in contact with human feces, including through sexual contact (*2*). Infection can also occur through contaminated food and water (*3*,*4*) or travel to endemic countries (*5*). Community outbreaks are associated with childcare settings, schools, residential institutions, and restaurants (*6*–*8*). Persons at highest risk for infection include those attending childcare settings, travelers to endemic countries, and gay, bisexual, and other men who have sex with men (GBMSM).

The implementation of whole-genome sequencing (WGS) for public health surveillance of bacterial pathogens has enabled global monitoring of the emergence and transmission of epidemic strains of *Shigella* spp. and antimicrobial resistance. Antimicrobial-resistant *S. sonnei* were first described >60 years ago (*9*–*11*), and multidrug-resistant (MDR) strains resistant to aminoglycosides, sulphonamides, trimethoprim, or chloramphenicol are endemic in the human population on every continent (*12*). Resistance to fluroquinolones has recently emerged, including from regions where antimicrobial use is unregulated (*13*–*15*). The increasing incidence of MDR and extensively drug-resistant (XDR) shigellosis in high-prevalence regions where surveillance is limited can be monitored by sequencing strains of *S. flexneri* and *S. sonnei* isolated from returning travelers and analyzing the genome derived antimicrobial resistance profiles.

Since 2010, surveillance systems maintained by the United Kingdom Health Security Agency (UKHSA) have identified a series of epidemics of MDR *S. flexneri* serotypes 3a, 2a, and 1b and *S. sonnei* among GBMSM; the strains are circulating nationally and internationally (*12*,*16*–*18*). Previous studies have demonstrated the acquisition of a plasmid encoding resistance to macrolides corresponded with the emergence of epidemics of *S. flexneri* 3a and 2a and *S. sonnei* during 2010–2015 (*17*,*19*). The subsequent global increase in notification of *S. sonnei* among GBMSM was enabled by strains belonging to global lineage 3.6.1.1.2 (clonal complex [CC] 152), exhibiting resistance to both macrolides and fluroquinolones (*16*). During the COVID-19 pandemic, a rapid decrease in notifications of *S. sonnei* was observed in the United Kingdom. However, after the relaxation of social distancing and travel restrictions, notifications quickly returned to prepandemic levels (*20*). We observed an increase in XDR *S. sonnei* with the *bla*_CTX-M-27_ gene variant conferring resistance to third-generation cephalosporins (*21*). Localized and short-lived outbreaks of XDR *S. sonnei* and *S. flexneri* containing the *bla*_CTX-M-27_ gene variant, primarily circulating within GBMSM sexual networks, were described previously (*18*,*22*). In contrast, an epidemic of sexually transmitted XDR *S. sonnei* was recorded in September 2021 (designated t10.377 by using the UKHSA single-linkage hierarchical clustering methodology, contained within global lineage 3.6.1.1.2 and CC152), continued into 2022 and was reported internationally (*21*).

After the publication of a study from France reporting an increase in the proportion of *Shigella* spp. isolates simultaneously resistant to ciprofloxacin, third-generation cephalosporins, and azithromycin (*23*), we reviewed genome-derived antimicrobial resistance profiles of the *S. sonnei* in the UKHSA archive isolated during 2016–2023. We identified an increasing trend of XDR strains of *S. sonnei* and found XDR *S. sonnei* isolated from MSM almost exclusively had the *bla*_CTX-M-27_ gene variant, whereas XDR *S. sonne*i isolated from travelers returning from high-risk regions almost exclusively had the *bla*_CTX-M-15_ gene variant (*24*). In 2023, we detected an increase of XDR *S. sonnei* in England that contained the *bla*_CTX-M-15_ gene variant. The aim of this study was to use a combination of epidemiologic data with short-read and long-read genomic sequencing data for outbreak investigation to determine emergence and transmission patterns of the *S. sonnei* outbreak strain and acquisition of the *bla*_CTX-M-15_ gene variant.

## Methods

### Routine Laboratory and Epidemiologic Surveillance

*Shigella* spp. isolates from hospital and community cases with gastrointestinal symptoms are referred to the gastrointestinal bacterial reference unit at the UKHSA for confirmation and typing. Since September 2015, we have conducted WGS for all *Shigella* isolates submitted to the gastrointestinal bacterial reference unit as previously described (*25*) and derived the serotype and antimicrobial resistance profile in silico from the genome. *S. sonnei* isolates submitted to the gastrointestinal bacterial reference unit during January 2016–December 2023 were included in this study. Because of the lack of sexual orientation information available in this dataset, we used a proxy indicator of cases that might be attributed to sexual transmission among GBMSM, defined as cases among male adults (>16 years) without a history of travel or where travel history was unknown (presumptive men who have sex with men [MSM]) (*26*).

We analyzed the sequencing data for genomic markers of resistance to azithromycin (defined as the presence of *ermB* or *mphA*), ciprofloxacin (defined as the presence of mutations in *gyrA*, *parC*, or *qnr*), and third-generation cephalosporins (defined by the presence of *bla*_CTX-M_ genes). We defined XDR isolates as those containing genomic markers of resistance to azithromycin, ciprofloxacin, and third-generation cephalosporins.

We conducted single-nucleotide polymorphism (SNP) typing on *S. sonnei* isolates. We applied single-linkage hierarchical clustering at 7 descending thresholds of SNP distances (Δ250, Δ100, Δ50, Δ25, Δ10, Δ5, Δ0) as previously described (*26*). That clustering resulted in a discrete 7-digit code in which each number represents the cluster membership at each descending SNP distance threshold. For *Shigella* spp. surveillance, we designated isolates that cluster at the 10 SNP threshold t10.X. We duplicated sequencing data in line with routine genomic surveillance of *Shigella* spp. at UKHSA. We tested the differences in proportions by using 2-proportion Z-tests and defined p<0.05 as significant.

### Phylogenetic Tree Construction

We used the WGS data from routine laboratory surveillance to create a phylogenetic tree of *S. sonnei* isolates with *bla*_CTX-M_ gene variants. We produced 1,325 samples from a soft-core genome alignment of CC152 within nucleotide cluster t25:1, in which a given variant position belonged to <80% of strains in the alignment, by using SnapperDB v0.2.8 (*27*). We previously masked recombinant sequences from a whole genome alignment derived from SnapperDB v0.2.8 (*27*) on the same dataset by using Gubbins v3.2 (*28*). We used the alignment (2,142,354 bp) as the input for IQ-TREE v2.0.4 (*29*) to generate a phylogenetic tree. We then repeated the methodology to produce subtrees of each cluster containing genomes with *bla*_CTX-M_ variants. For each phylogeny, the tree was rooted by the most closely related strain outside the cluster range in question.

### Nanopore Sequencing and De Novo Assembly

We used Illumina (Illumina, https://www.illumina.com) for routine sequencing and Oxford Nanopore (Oxford Nanopore Technologies, https://nanoporetech.com) for long-read sequencing to generate complete assemblies of selected *bla*_CTX-M_ variant samples to understand the genetic context for antimicrobial resistance determinants. We extracted and sequenced genomic DNA by using the MinION (Oxford Nanopore Technologies) and processed data, trimmed reads, and assembled as described previously (*30*).

We conducted de novo assembly by using Flye v2.9.2 (*31*). We corrected the assemblies by using Medaka version 1.0.3 (https://github.com/nanoporetech/medaka) with a *Shigella*-specific medaka-trained model, and then by using Polypolish v0.5.0 (*32*) with the equivalent Illumina FASTQs (Illumina) for each assembly. Because all the contigs were circular and closed, we reoriented them to start at the *dnaA* gene (GenBank accession no. NC_000913) from *E. coli* K12, by using the fix start parameter in Circlator version 1.5.5 (*33*).

### Antimicrobial Resistance Gene Detection and Plasmid Typing

We detected the plasmid replicon for each nonchromosomal contig within the final assembly of each sample by using PlasmidFinder version 2.1 (*34*) with the Enterobacteriaceae database and these parameters: minimum identity = 90% and minimum coverage = 90%. We annotated the mobile genetic elements with antimicrobial resistance determinants by using the Prokaryotic Genome Annotation Pipeline build 2022-12-13 (*35*). We generated gene-level alignments by using Clinker version 0.0.27 (*36*).

### Data Deposition

We submitted the FASTQ files and gene assemblies to the National Center for Biotechnology Information (BioProject no. PRJNA315192). Accession numbers have been provided (Appendix Table 1).

### Ethics Statement

This study was undertaken for health protection purposes. Permission was granted to UKHSA to collect and process confidential patient data under Regulation 3 of The Health Service (Control of Patient Information) Regulations 2020 and Section 251 of the National Health Service Act 2006.

## Results

### Descriptive Epidemiology

**Figure 1:**
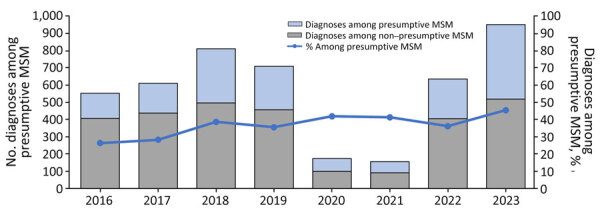
Epidemiologic and genomic investigation of sexually transmitted *Shigella sonnei* diagnoses in presumptive MSM classification, England, 2016–2023. Presumptive MSM category was defined as cases among male adults (>16 years of age) without a history of travel or where travel history was unknown. MSM, men who have sex with men.

*S. sonnei* diagnoses increased during 2016–2018, then declined slightly in 2019 and declined markedly in 2020 and 2021, likely because of reduced access to healthcare services and testing, social distancing, and travel restrictions during the COVID-19 pandemic (Figure 1). In 2022 and 2023, there was a substantial increase in *S. sonnei* diagnosis notifications, and the 2023 notifications exceeded prepandemic levels. The trends of *S. sonnei* among presumptive MSM mirror those among all persons. However, the rate of increase was larger among presumptive MSM, leading to an increase in the proportion of all *S. sonnei* diagnoses seen among presumptive MSM, from 26% in 2016 to 46% in 2023. The increase between 2022 and 2023 was also higher among presumptive MSM (82% increase) compared with all persons (50% increase).

**Figure 2:**
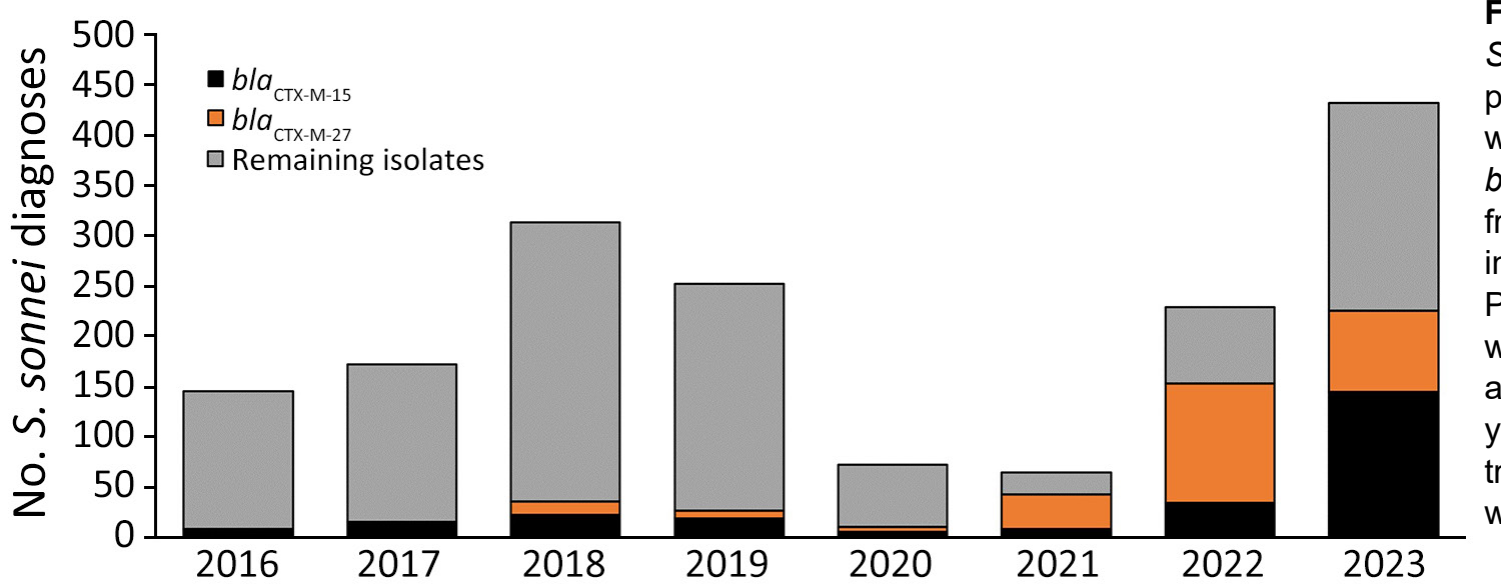
Sexually transmitted *Shigella sonnei* isolates among presumptive men who have sex with men by the presence of the *bla*_CTX-M-15_ or *bla*_CTX-M-27_ gene variant from an epidemiologic and genomic investigation, England, 2016–2023. Presumptive men who have sex with men category was defined as cases among male adults (>16 years of age) without a history of travel or where travel history was unknown.

Including the increase in *S. sonnei* among presumptive MSM in 2023, there was a corresponding increase in the number and proportion of *S. sonnei* isolates with the *bla*_CTX-M-15_ gene variant in the population. During 2016–2022, an average of 10% of *S. sonnei* isolates contained the *bla*_CTX-M-15_ gene variant, increasing to 33% in 2023. That increase in the proportion of *S. sonnei* isolates with the *bla*_CTX-M-15_ gene variant in 2023 corresponded with a decrease in *S. sonnei* isolates with the *bla*_CTX-M-27_ gene variant in this population group (Figure 2).

**Figure 3:**
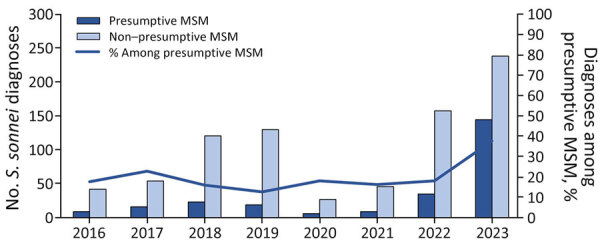
*Shigella sonnei* isolates with the *bla*_CTX-M-15_ gene variant among presumptive men who have sex with men compared with nonpresumptive men who have sex with men from an epidemiologic and genomic investigation, England, 2016–2023. Presumptive MSM was defined as cases among male adults (>16 years of age) without a history of travel or where travel history was unknown. MSM, men who have sex with men.

Before 2023, *S. sonnei* isolates with the *bla*_CTX-M-15_ gene variant were identified at a much lower frequency among presumptive MSM compared with non–presumptive MSM (i.e., women, children, and men reporting recent travel). During 2016–2022, the proportion of *S. sonnei* with the *bla*_CTX-M-15_ gene variant among presumptive MSM remained stable at an average of 17%, increasing to 38% in 2023 (Figure 3).

### Phylogenetic Analysis of *S. sonnei* with the *bla*_CTX-M-15_ Gene Variant

**Figure 4:**
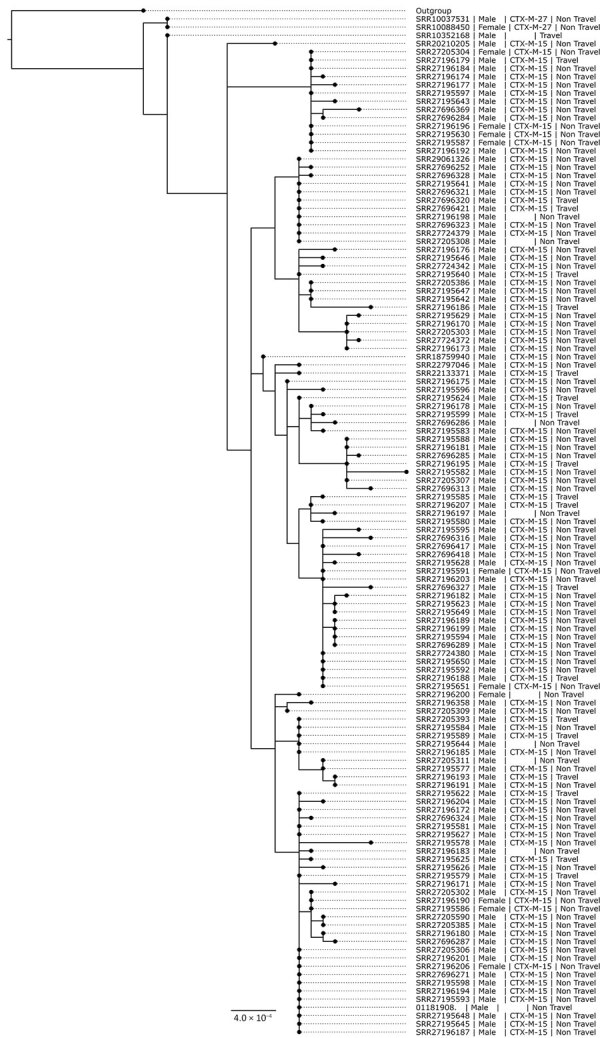
Maximum-likelihood phylogenetic tree of the *Shigella sonnei* clonal complex 152 within the 10 single-nucleotide polymorphism linkage cluster t10.1814 (n = 125) found during an epidemiologic and genomic investigation of sexually transmitted *S. sonnei* from presumptive men who have sex with men, England, 2016–2023. A total of 125 isolates included in tree. Sequence read run accession is sample identification, additional information provided is gender, presence of *bla*_CTX-M_ gene variants, and association with travel. Presumptive men who have sex with men was defined as cases among male adults (>16 years of age) without a history of travel or where travel history was unknown.

**Figure 5:**
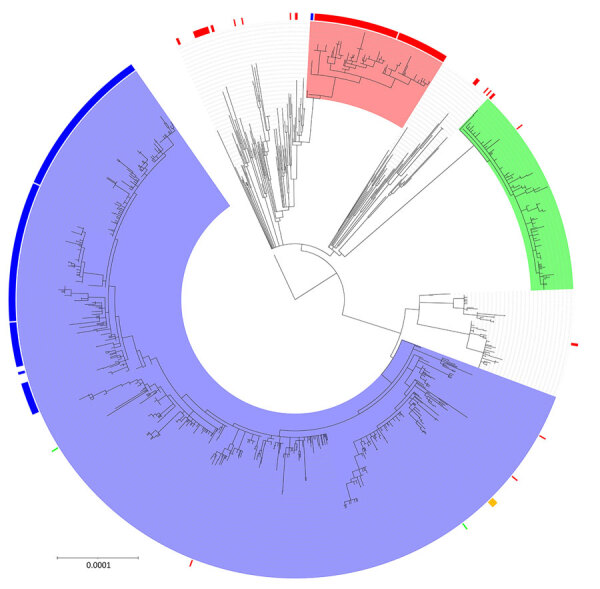
Maximum-likelihood phylogenetic tree of clonal complex 152 within nucleotide cluster t25:1 found during an epidemiologic and genomic investigation of sexually transmitted *Shigella sonnei* from presumptive men who have sex with men, England, 2016–2023. Included in the tree were 1,325 isolates. Blue, t10.377 cluster; green, t10.2218 cluster; red, t10.1814. The outer ring indicates presence of *bla*_CTX-M_; red, *bla*_CTX-M-15_; blue, *bla*_CTX-M-27_; orange, *bla*_CTX-M-134_; green, *bla*_CTX-M-55._ Presumptive men who have sex with men was defined as cases among male adults (>16 years of age) without a history of travel or where travel history was unknown.

Of the 262 *S. sonnei* isolates with the *bla*_CTX-M-15_ gene variant collected during 2016–2023 from presumptive MSM, 84 (32%) fell within a 10-SNP single linkage cluster (SCL) designated t10.1814 (full SNP address 1.1.1.1.1814) and belonging to global lineage 3.6.1.1 (*37*) (Figure 4). In addition, 2 other 10-SNP SCLs contained isolates with the *bla*_CTX-M-15_ gene variant were identified, t10.1148 (full SNP address 1.1.29.49.1148) and t10.2187 (full SNP address 1.1.29.49.2187). Those 2 clusters fall within the same 25-SNP SCL (t25:49), a lineage that is associated with travel to Pakistan (82% of case-patients reporting travel to Pakistan) and Tunisia (94% of case-patients reporting travel to Tunisia), but distinct from the 25-SNP SCL containing t25:1. The remaining *S. sonnei* isolates with the *bla*_CTX-M-15_ gene variant from presumptive MSM were phylogenetically sporadic and did not form large clusters (Figure 5). Therefore, our phylogenetic analysis focuses on the t10.1814 cluster to explore the increase in *S. sonnei* containing the *bla*_CTX-M-15_ gene variant among presumptive MSM (Figure 4).

**Figure 6:**
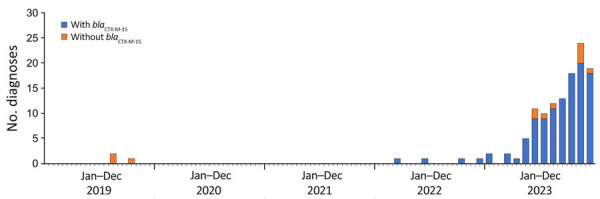
Diagnoses of *Shigella sonnei* in cluster t10.1814 by the *bla*_CTX-M-15_ gene variant status from an epidemiologic and genomic investigation, England, 2016–2023.

At the end of 2023, the t10.1814 cluster contained 124 isolates in total. The first 3 cases within the cluster were diagnosed in August and October 2019. None of those isolates contained *bla*_CTX-M-15_; however, 2 of the 3 isolates contained the *bla*_CTX-M-27_ gene variant (Figures 4,5,6). There was no reported activity within the t10.1814 cluster until March 2022, but 4 cases were reported during March–December 2022. There was a substantial increase in cases in 2023, and most isolates (94%, 117/124) in the cluster had specimen dates in 2023. Of the 2023 isolates, 92% (108/117) contained the *bla*_CTX-M-15_ gene variant (Figure 6). Of the 124 cases in the cluster, 75% (n = 93) were adult men with no or unknown travel, 16% (n = 20) were adult men with travel outside the UK (mostly to countries in Europe), and 9% (n = 11) were women or children with no or unknown travel (Table 1).

**Table 1:** Characteristics of sexually transmitted *Shigella sonnei* from an epidemiologic and genomic investigation of the t10.1814 cluster, England, 2016–2023*

Characteristics	Cases, N = 124
Sex	
M	113 (91)
F	11 (9)
Age, y	
Median [IQR]	35 [30–43]
Adult, >16	123 (99)
Child, <16	1 (1)
Travel	
Yes	20 (16)
No/Unknown	104 (84)
Travel destination of those reporting travel	
Belgium	1 (5)
Brazil	1 (5)
Europe, country unknown	1 (5)
Germany	1 (5)
India	1 (5)
Netherlands	1 (5)
North America	1 (5)
Spain	8 (40)
Thailand	1 (5)
United states	1 (5)
Unknown	3 (15)
Presumptive MSM	
Yes	93 (75)
No	31 (25)

*Values are no. (%) except as indicated. Presumptive MSM category was defined as cases among male adults (>16 years of age) without a history of travel or where travel history was unknown. MSM, men who have sex with men.

**Figure 7:**
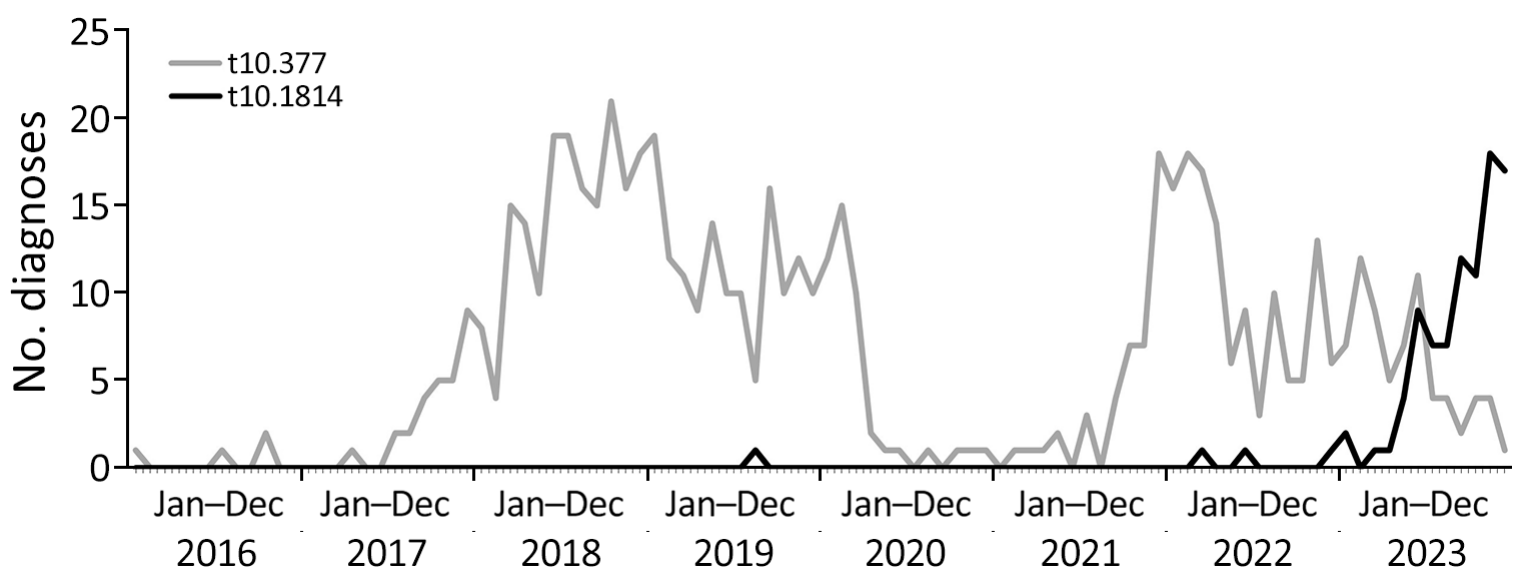
Diagnoses of *Shigella sonnei* in the t10.377 cluster compared with the t10.1814 cluster among presumptive men who have sex with men from an epidemiologic and genomic investigation, England, 2016–2023. Presumptive MSM was defined as cases among male adults (>16 years of age) without a history of travel or where travel history was unknown.

The increase in t10.1814 occurred in parallel to a decline in cases within the 1.1.1.1.377.% cluster (designated t10.377; the % indicates that t3 and t0 positions of the SNP address can take any value). The cluster was dominant since 2017, declined substantially during the first few months of the COVID-19 pandemic, but reemerged in September 2021 with third-generation cephalosporin resistance caused by the *bla*_CTX-M-27_ gene variant (*21*) (Figure 7). Despite the different trends in those clusters, t10.1814 and t10.377 share similarities in demographic characteristics of cases; the t10.377 cluster was also associated with presumptive MSM (Appendix Table 2).

Of the 124 isolates in the t10.1814 cluster, 112 (90%) harbored the *bla*_CTX-M-15_ gene variant, whereas only 2 isolates had the *bla*_CTX-M-27_ gene variant; no isolates expressed both the *bla*_CTX-M-15_ and *bla*_CTX-M-27_ gene variants. Resistance to ciprofloxacin and azithromycin was very high; 94% of isolates (116/124) exhibited mutations in either *gyrA*, *parC*, or the plasmid mediated *qnr* gene variant, and 88% of isolates (109/124) had genomic markers for azithromycin resistance (*ermB* or *mphA*). Most (86%) isolates had both the *bla*_CTX-M-15_ gene variant and markers of azithromycin resistance. Overall, 109 (88%) isolates in the t10.1814 cluster were XDR (Table 2).

**Table 2:** Antimicrobial resistance profile of sexually transmitted cases of *Shigella sonnei* from an epidemiological and genomic investigation of cases within the t10.1814 cluster, England, 2016–2023*

Antimicrobial and resistance determinant	Cases, N = 124
Third-generation cephalosporin	
*bla*_CTX-M-15_ gene variant	112 (90)
*bla*_CTX-M-27_ gene variant	2 (2)
Ciprofloxacin: *gyrA*, *parC*, or *qnr*	116† (94)
Azithromycin: *ermB* or *mphA*	109 (88)
Extensively drug resistant	109 (88)

*Values are no. (%). †All isolates had 2 point mutations in *gyrA* and 1 point mutation in *parC*.

Phylogenetic analysis revealed that t10.1814 fell within the wider t25:1 cluster that also includes t10.377. Although located within the same t25 SLC, the t10.1814 cluster with *bla*_CTX-M-15_ was located on a separate branch and did not evolve from the t10.377 cluster with the *bla*_CTX-M-27_ gene variant (Figure 5). The progenitor strains of t10.1814 clustered with the *bla*_CTX-M-15_ gene variant also contained the *bla*_CTX-M-27_ gene variant.

### Analysis of t10.1814 IncFII Plasmids and Comparison to t10.377 IncFII Plasmids

**Figure 8:**
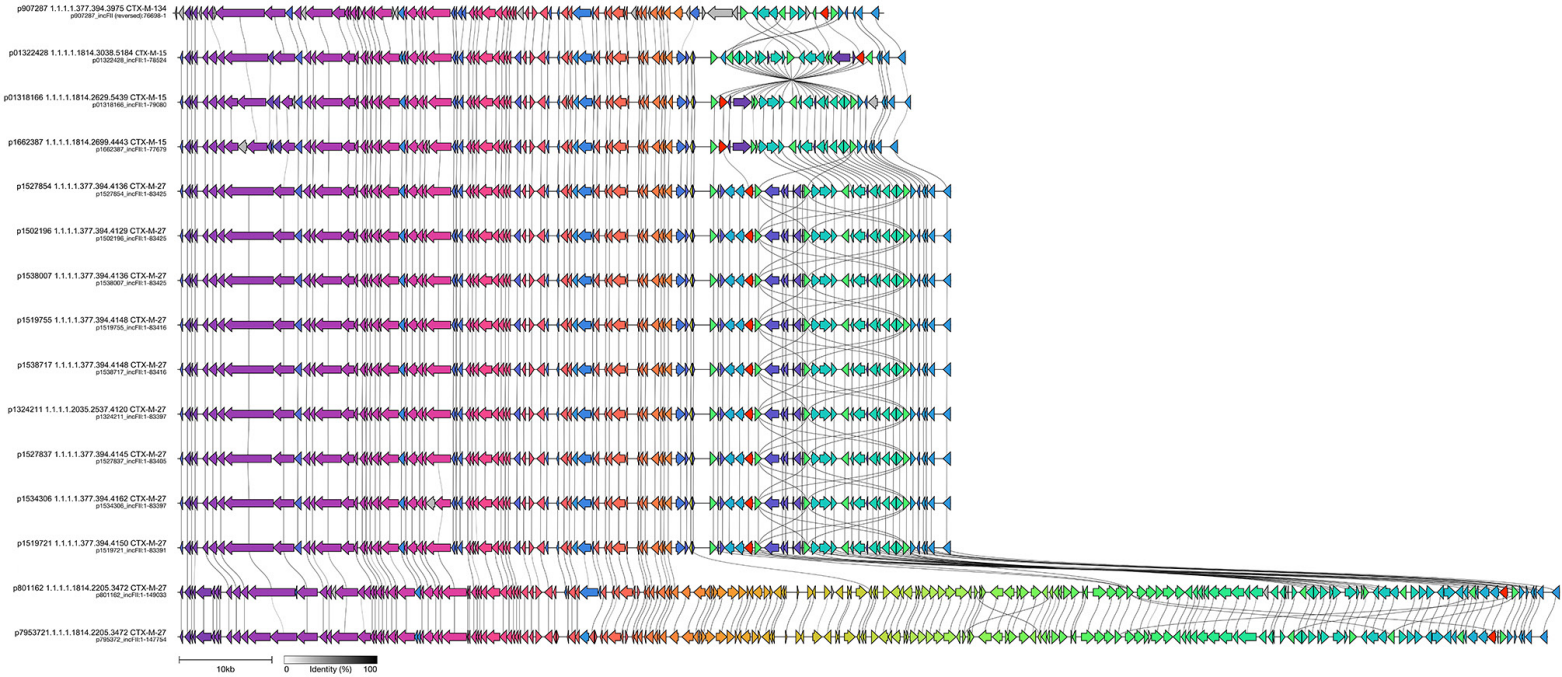
Alignment of IncFII plasmids in samples selected for Nanopore sequencing during an epidemiologic and genomic investigation of sexually transmitted *Shigella sonnei* from presumptive men who have sex with men, England, 2016–2023. Red gene is the *bla*_CTX-M_ variant. Presumptive men who have sex with men was defined as cases among male adults (>16 years of age) without a history of travel or where travel history was unknown.

Plasmids within t10.1814 with the *bla*_CTX-M-15_ gene variant were all determined to be of the IncFII replicon type and ranged from 77.6 to 149.0 kbp in size (Figure 8). Plasmids from progenitor strains within t10.1814 with the *bla*_CTX-M-27_ gene variant were larger on average (148 kbp) than plasmids with the *bla*_CTX-M-15_ gene variant (78.4 kbp). Despite the difference in size, almost all the gene content of the ≈74 kbp plasmids were also found within the larger ≈148 kbp plasmids. Of note, when comparing t10.1814 plasmids to IncFII plasmids from t10.377, the plasmid structure is the same except for small alterations to the variable region, including the *bla*_CTX-M-15_ and *bla*_CTX-M-27_ integrons (Figure 8).

**Figure 9:**
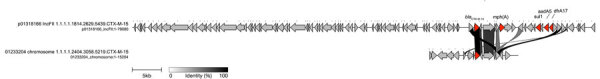
Alignment of exemplar IncFII plasmid from a *Shigella sonnei* strain during an epidemiologic and genomic investigation of sexually transmitted *Shigella sonnei* from presumptive men who have sex with men, England, 2016–2023. The strain fell within the 10 single-nucleotide polymorphism linkage cluster t10.1814 and strain 01233204 (GenBank accession no. SRR29176725), showing the cassette containing *bla*_CTX-M-15_ (highlighted in red) has moved to the chromosome. Presumptive men who have sex with men was defined as cases among male adults (>16 years of age) without a history of travel or where travel history was unknown.

After the analysis of the long-read sequencing data, we identified 1 isolate of *S. sonnei* with the *bla*_CTX-M-15_ gene variant in the same clade as t10.1814 but in a different t10 SLC (t10.2404). In that cluster, the *bla*_CTX-M-15_ gene variant was located on a 7.6-kbp cassette or an element integrated on the chromosome. The integration site appears to be a prophage remnant near a tRNA-Phe gene (Figure 9). The IncFII plasmid was lost in this sample.

## Discussion

Overall, except for 2020 and 2021 when notifications were affected by the COVID-19 pandemic, we observed a steady increase of *S. sonnei* diagnoses in England during 2016–2023. During the past decade, diagnostic methods for the detection of gastrointestinal pathogens have improved with widespread implementation of commercial PCRs. PCR is more sensitive than culture for detecting *Shigella* spp. (*38*,*39*), and this move toward molecular methods after culture will increase case confirmation. Furthermore, the increase might be associated with increased travel to high-risk regions outside the United Kingdom, although confirming that theory is difficult because travel history is poorly captured by the current surveillance system. We also observed a steady increase in notifications of *S. sonnei* among presumptive MSM. Although our observation may reflect a true increase in sexual transmission, it might also be influenced by increased implementation of PCR testing and travel. The increase in reported diagnoses might be because of the publication of briefing notes and other outbreak-related communications by UKHSA during the study period.

Numerous factors enable the emergence, transmission, and persistence of epidemic strains circulating within GBMSM sexual networks, involving pathogen characteristics, host behaviors, and environmental pressures. We have previously hypothesized that the sequential waves of shigellosis among GBMSM in the United Kingdom have been enabled by acquisition of antimicrobial resistance to an increasing number of classes of antimicrobial drugs (*40*). The epidemic strains of *S. sonnei* were initially resistant to macrolides, then to both azithromycin and ciprofloxacin, and most recently to macrolides, fluroquinolones, and third-generation cephalosporins (*16*,*21*). However, the acquisition of antimicrobial resistance alone does not explain the emergence and persistence of all shigellosis epidemics among GBMSM. In this study, we showed the previous epidemic *S. sonnei* strain (t10.377) was replaced by another strain of *S. sonnei* (t10.1814) with the same genotypic antimicrobial resistance profile, and the reemergent strain of *S. flexneri* 3a in 2019 was more susceptible to antimicrobials than the strain that caused the original *S. flexneri* 3a epidemic (*41*). Asymptomatic transmission of *Shigella* spp. among GBMSM might be a factor driving antimicrobial pressure in this group (*42*). Other strains during previous epidemics are examples of the emergence and persistence of strains exhibiting the same antimicrobial resistance profiles. Other factors could be at play, such as transient host immunity to circulating serotypes providing emergent serotypes with a competitive advantage. Host immunity seems an unlikely explanation for the strain replacement event because both strains were *S. sonnei*.

Overall, the case characteristics in the t10.1814 cluster were similar to those in the t10.377 cluster in terms of the proportion of male cases and age distribution. Some regional variation exists; cases in the t10.1814 cluster were more dispersed across regions of England, and the t10.377 cluster was more concentrated in London (Appendix Table 2). The difference in travel history between cases in the clusters could be because of missing data on recent travel history.

Phylogenetic analyses showed clustering of *bla*_CTX-M_ variants within the *Shigella* spp. population structure, consistent with horizontal acquisition and vertical transmission. Non-GBMSM clades associated with the *bla*_CTX-M-15_ gene variant comprised cases reporting travel to high-risk regions outside the United Kingdom, highlighting the possibility that this resistance determinant was brought in through travel, similar to *Shigella* in other regions (*43*,*44*). One GBMSM *bla*_CTX-M-15_ gene variant isolate fell within the same 10 SNP SLC, and although the *bla*_CTX-M-27_ gene variant decrease coincided with the *bla*_CTX-M-15_ gene variant increase, there was no evidence the *bla*_CTX-M-15_ gene variant emerged from the clade with the *bla*_CTX-M-27_ gene variant. The acquisition of the *bla*_CTX-M-15_ gene variant appears to be an independent evolutionary event on a different branch of the phylogeny.

Long-read sequencing analysis revealed that, like the *bla*_CTX-M-27_ gene variant in the t10.377 cluster, the *bla*_CTX-M-15_ gene variant in the current epidemic t10.1814 cluster was located on an IncFII plasmid. Despite encoding different *bla*_CTX-M_ variants, the plasmid encoding the *bla*_CTX-M-15_ gene variant exhibited high levels of similarity to the plasmid encoding the *bla*_CTX-M-27_ gene variant. Those data reveal similar IncFII plasmids persist and remain stable in the strains of *S. sonnei* circulating among GBMSM, despite acquisition of different antimicrobial resistance determinants. Because of the apparent plasmid stability in this population, our demonstration of the acquisition of the *bla*_CTX-M-27_ and *bla*_CTX-M-15_ gene variants and subsequent clonal expansion, the potential other antimicrobial resistance determinants could be acquired onto this plasmid and worsen the already concerning antimicrobial resistance picture of *S. sonnei* remains. In addition, we report an isolate in a separate clade (t10.2404) in which the *bla*_CTX-M-15_ gene variant was located on the chromosome and the associated plasmid was lost.

Social distancing and travel restrictions in 2020 and 2021 related to the COVID-19 pandemic had a greater effect on reducing notifications of *S. sonnei* than *S. flexneri* (*25*). Previously, we considered that globalization and increased travel might have a role in seeding sexually transmissible shigellosis. The acquisition of the *bla*_CTX-M-15_ gene variant previously associated with travel-related cases of *S. sonnei*, on the GBMSM-associated IncFII pKSR-100-like plasmid, may provide further evidence for this hypothesis. The reporting of *S. sonnei* with the *bla*_CTX-M-15_ gene variant among GBMSM in other countries in Europe suggests the potential international distribution of this lineage (*45*,*46*).

With a lack of information about sexual orientation and incomplete travel histories, it is possible that adult male case-patients who traveled were categorized as presumptive MSM within this cluster if the travel histories were not known. Identifying as GBMSM and reporting recent travel are also not mutually exclusive; therefore, there are limitations with the use of the presumptive MSM proxy definition. It is also not mandatory for primary diagnostic laboratories to send *S. sonnei* isolates to the gastrointestinal bacterial reference unit, so the data available for this analysis represents about two thirds of the total number of reported infections.

Despite those limitations, the introduction of WGS for typing gastrointestinal pathogens greatly improved surveillance of *S. sonnei* at UKHSA. Previously, we relied on phenotypic methods that were highly specialized, labor intensive, and difficult to standardize, such as phage typing and antimicrobial susceptibility testing. During the past decade, sequencing data has been used to construct the population structure of *S. sonnei* from UK residents and mapped clades associated with travel and associated with sexual transmission among GBMSM. We have tracked the rise and fall of different clades circulating within GBMSM sexual networks and showed that acquisition of antimicrobial resistance and genetic factors contribute to emergence, transmission, and persistence. However, notifications continue to rise, and the circulating strains are increasingly resistant to first- and second-line antimicrobial drugs.

The results in this article highlight the continued utility of genomic surveillance in detecting outbreaks of sexually transmissible shigellosis and the ever-growing importance of antimicrobial stewardship for shigellosis (*47*). Furthermore, through detailed analyses of the data, we can clarify the complex origins and transmission pathways for antimicrobial resistance in increasingly antimicrobial-resistant strains. We recurrently see conjugative plasmids carrying resistance against key antimicrobial classes mobilizing among *Shigella* spp. strains circulating in different transmission networks. This plasmid mobilization underlines the need to address *Shigella* spp. as an urgent antimicrobial threat, in line with the World Health Organization priority pathogen list of 2024 (*48*), and highlights the need to create innovative solutions to slow sexual transmission in networks in which heavy antimicrobial use drives the emergence of XDR strains.

Appendix Additional information about epidemiologic and genomic investigation of sexually transmitted *Shigella sonnei*, England.
